# Personalized Computational Models as Biomarkers

**DOI:** 10.3390/jpm7030009

**Published:** 2017-09-01

**Authors:** Walter Kolch, Dirk Fey

**Affiliations:** 1 Systems Biology Ireland, University College Dublin, Belfield, Dublin 4, Ireland; 2 Conway Institute of Biomolecular & Biomedical Research, University College Dublin, Belfield, Dublin 4, Ireland; 3 School of Medicine, University College Dublin, Belfield, Dublin 4, Ireland

**Keywords:** biomarkers, neuroblastoma, mathematical/computational modelling, personalized medicine

## Abstract

Biomarkers are cornerstones of clinical medicine, and personalized medicine, in particular, is highly dependent on reliable and highly accurate biomarkers for individualized diagnosis and treatment choice. Modern omics technologies, such as genome sequencing, allow molecular profiling of individual patients with unprecedented resolution, but biomarkers based on these technologies often lack the dynamic element to follow the progression of a disease or response to therapy. Here, we discuss computational models as a new conceptual approach to biomarker discovery and design. Being able to integrate a large amount of information, including dynamic information, computational models can simulate disease evolution and response to therapy with high sensitivity and specificity. By populating these models with personal data, they can be highly individualized and will provide a powerful new tool in the armory of personalized medicine.

## 1. Introduction: The Role of Biomarkers

Biomarkers are a pillar of modern medicine. They are used to identify people at risk of diseases, diagnose diseases, choose treatments and monitor the success of treatments. There are many definitions of biomarkers [1], but they all share the notion that they can objectively assess, quantify, and sometimes prognosticate the medical state of a patient independent of personal symptoms experienced by the patient. This definition is increasingly broadening to take into account not only changes in patients’ internal parameters but also interaction with external parameters, such as treatment, lifestyle and environmental exposure. This expansion is largely founded in the recognition that disease is a highly dynamic process whose origin and course is subject to multiple influences. Biomarkers which can capture these multidimensional features will be necessary to realise the potential of personalised medicine, which relies on the precise stratification of patients and their disease for prevention and diagnosis. Much hope rests on new technologies, such as genome sequencing, helping us to understand the complex interactions between disease and the human organism. So, where are we in terms of such biomarkers?

## 2. The Limitations of Current Biomarkers

Although classic biomarkers have been invaluable tools in the arsenal of clinical diagnostics, most of them tell surprisingly little about pathogenetic mechanisms. For instance, high blood pressure indicates that the cardiovascular system is compromised and will eventually fail, but it does not reveal why and how. Thus, a major shortcoming of existing biomarkers is that they usually do not reveal any information about pathogenetic mechanisms. A biomarker even may provide misleading comfort of understanding the pathogenetic mechanisms of a disease by classifying the disease. An example is high blood glucose, which defines diabetes—but without revealing anything about its pathogenesis. Thus, great efforts have been invested in finding new and better biomarkers, increasingly making use of global molecular profiling methods, such as genome sequencing, transcriptomics, proteomics and metabolomics. So far, results have been somewhat disappointing. Genome sequencing discloses thousands of genetic variants, the meaning of which is largely unknown. Moreover, only few diseases can be traced to aberrations in a single gene. Many of the common diseases are multifactorial and their complexity makes it difficult to unravel the combinatorial influences exerted by genetic factors. Moreover, even where genetic changes can indicate a disease predisposition, they usually cannot tell when the risk will manifest itself and who will actually be affected. More dynamic methods, especially transcriptomics and proteomics, have provided a better stratification in this respect. For instance, several transcriptomics based assays, such as Oncotype DX (Genomic Health, Inc. Redwood City, CA, USA) and Mammaprint (Agendia NV, Amsterdam, The Netherlands), are being used to assess the prognosis and possible requirement of chemotherapy treatment in breast cancer patients [2]. A common lesson from these efforts was that it usually requires an ensemble of biomarkers, a biomarker signature, to achieve diagnostic accuracy. How can we improve on that?

## 3. Mathematical and Computational Models as Biomarkers

Mathematical and computational modelling has been successfully used to investigate the dynamic behaviour of biological systems, especially the signal transduction networks (STNs) that govern fundamental cellular processes such as the cell cycle, cell growth, cell death, inflammation and processes disturbed by disease [3]. These models allow the systematic assessment of STN perturbations and adaptive responses through in silico simulations. STNs are altered in virtually any disease, and therefore provide attractive avenues to find or even become new biomarkers. We have recently delivered a proof of concept study in neuroblastoma, a childhood tumour of extremely heterogeneous prognosis [4]. Neuroblastoma is treated by surgery followed by genotoxic chemotherapy, which can have severe long-term side effects. Thus, accurate patient stratification is imperative [5]. About 20% of neuroblastomas feature an amplification of the *MYCN* gene, which is associated with poor prognosis demanding aggressive chemotherapy. However, there is no biomarker for poor prognosis neuroblastoma without *MYCN* amplification. Using an integrated experimental approach that mapped the response of neuroblastoma cells to chemotherapeutic agents, we identified the stress activated JUN N-terminal kinase (JNK) STN as critical for mediating response to therapy [4]. Generating a mathematical model allowed us to identify the three critical control nodes of the STN that determine the dynamic response to chemotherapy. By measuring these three control nodes in patient samples and substituting the values in the generic model, we could develop personalised models for each patient. The analysis of two independent patient cohorts showed that the model could accurately identify high risk patients independently of *MYCN* amplification. Thus, the development of personalised, STN based disease models with accurate and prognostic value is highly feasible. Notably, modelling of metabolic networks supports the value of dynamic modelling over correlative biomarker signatures. For instance, recent work on erythrocyte metabolism showed that personalised dynamic models correspond to genotypes better than metabolite levels, and can identify individuals who may suffer drug side effects [6]. Large-scale community efforts are underway to develop the potential of metabolic pathway modelling for biomarker and drug target discovery efforts [7].

## 4. Future Outlook

Our neuroblastoma study has demonstrated that dynamic mathematical models can serve as highly useful biomarkers for personalised medicine. A unique advantage of this approach is that the models can identify STN control nodes. This is an important distinction versus biomarker signatures, which identify correlations rather than causalities. This has important implications. First, dynamic mathematical models integrate a huge amount of information, which is preserved in the output of the computational simulation; second, they can account for dynamic adaptations, making them useful for predicting when a risk will manifest itself and how it will respond to therapy; third, the STN model analysis reveals key pathogenetic mechanisms, i.e., the aberrations in STNs that determine disease progression and aggressiveness. Fourth, the identification of STN control nodes also provides concrete targets for therapeutic intervention as control nodes not only reflect the state of a system but are also natural points for manipulating it. The latter point is of particular interest considering that it provides accurate diagnostics and therapeutic targets at the same time. This may solve the longstanding dilemma of developing companion diagnostics for new drugs. Studies have shown that drugs with companion diagnostics have a higher chance to succeed in clinical trials [8]. Thus, computational models kill two birds with one stone and may help to relieve the unsustainable attrition rate of drugs making it to the patient. While sparse patient derived data were successfully used to establish prognostic correlations [9,10], dynamic modelling can add much needed causal relationships.
